# Newspaper Adherence to Media Reporting Guidelines for the Suicide Deaths of Kate Spade and Anthony Bourdain

**DOI:** 10.1001/jamanetworkopen.2019.14517

**Published:** 2019-11-01

**Authors:** Arielle H. Sheftall, Jaclyn L. Tissue, Paige Schlagbaum, Jonathan B. Singer, Nerissa Young, Jack H. Stevens, John P. Ackerman

**Affiliations:** 1 Department of Pediatrics, The Abigail Wexner Research Institute at Nationwide Children’s Hospital, The Ohio State University College of Medicine, Columbus; 2 Center for Innovation in Pediatric Practice, The Abigail Wexner Research Institute at Nationwide Children’s Hospital, Columbus, Ohio; 3 School of Social Work, Loyola University Chicago, Chicago, Illinois; 4 E.W. Scripps School of Journalism, Ohio University, Athens; 5 Center for Biobehavioral Health, Nationwide Children’s Hospital, Columbus, Ohio; 6 Department of Psychiatry and Behavioral Health, The Ohio State University College of Medicine, Columbus; 7 Department of Behavioral Health, Nationwide Children’s Hospital, Columbus, Ohio

## Abstract

This cross-sectional study examines newspaper adherence to reporting guidelines for suicide after the deaths of Kate Spade and Anthony Bourdain.

## Introduction

News media coverage of suicide is associated with an increased risk of subsequent suicides, with the strongest associations following newspaper reporting of celebrity suicides.^1^ To reduce these adverse effects, media guidelines were established for reporting on suicide in 2001^2^; however, adherence varies, and research shows many media outlets are unaware that such guidelines exist.^3^

On June 5, 2018, Kate Spade died by suicide, and on June 8, 2018, Anthony Bourdain died by suicide. These events provided an opportunity to examine newspaper adherence to reporting guidelines. Because much criticism followed the reporting on Spade’s death,^4^ we hypothesized that the reporting on Bourdain’s death would be more guideline adherent.

## Methods

**Figure 1.**
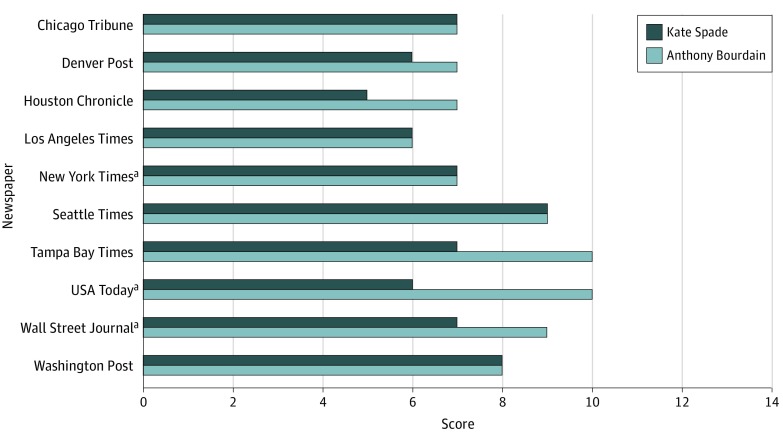
Reporting Guideline Adherence Scores for Select US Newspapers Covering the Deaths of Spade and Bourdain Scores could range from 0 to 14; a score of 12 or higher was set as the threshold for high fidelity. A paired *t* test for differences revealed significantly higher mean (SD) adherence scores for newspaper reporting on the suicide death of Anthony Bourdain compared with reporting on the suicide death of Kate Spade (8.0 [1.4] vs 6.8 [1.1]; mean difference = 1.2; 95% CI, 0.14-2.26; *t*_9_ = 2.57; *P* = .03). ^a^National newspaper.

Newspapers were selected based on US geographic regions and daily circulation data.^5^ All 4 US regions (ie, Northeast, South, Midwest, and West) were represented, with no more than 2 newspapers from the same city. The 10 print newspapers included 3 national and 7 regional newspapers (Figure 1), all with a minimum circulation of 200 000.^5^ Articles about Spade and Bourdain were obtained for June 6, 2018, and June 9, 2018, respectively; June 11, 2018, was used for Bourdain’s death for *USA Today* because this newspaper publishes on weekdays only.

Three of us (J.B.S., N.Y., and J.P.A.) evaluated each article to determine adherence to reporting guidelines. Newspaper names were removed to eliminate potential bias. However, headlines, pictures and captions, and location in the newspaper were provided. Each reviewer received articles in a randomized order to avoid order effects.

Adherence was assessed using 14 variables derived from recommendations from Reporting on Suicide and the American Foundation for Suicide Prevention.^2^ Final consensus occurred via conference call. Interrater agreement averaged across the individual guidelines prior to consensus was deemed excellent (Cohen κ = 0.893; range, 0.705-1.000). Adherence was calculated for each article, with scores of at least 12 (indicating ≥80% adherence) set as the high-fidelity threshold, per the approach of Creed and Whitley.^6^

A paired *t* test was conducted to compare adherence scores for reporting on both deaths. Statistical significance was set at *P* < .05, and all tests were 2-tailed. Statistical analysis was conducted with SPSS statistical software version 25 (IBM Corp). This study complied with Strengthening the Reporting of Observational Studies in Epidemiology (STROBE) reporting guideline for cross-sectional studies. The study was not considered human subjects research according to the review policy of The Abigail Wexner Research Institute at Nationwide Children’s Hospital institutional review board.

## Results

Figure 1 displays the guideline adherence scores of each newspaper. Overall, newspapers adhered to a mean (SD) of 7.4 (1.4) of 14 specific guidelines; none reached the threshold for high fidelity. A paired *t* test revealed a higher mean (SD) score for reporting on Bourdain’s death compared with Spade’s death (8.0 [1.4] vs 6.8 [1.1]; mean difference = 1.2; 95% CI, 0.14-2.26; *t*_9_ = 2.57; *P* = .03); this difference was associated with changes in scores for 5 of 10 newspapers.

**Figure 2.**
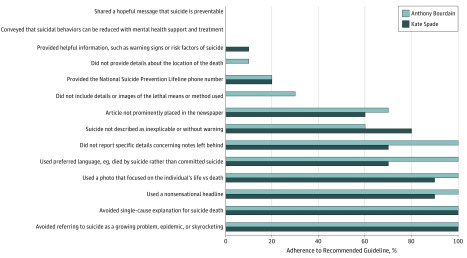
Percentage of Select US Newspapers Adhering to Specific Suicide Reporting Guidelines Guideline adherence was assessed using 14 items derived from recommended reporting guidelines on suicide. Each guideline was coded 1 for yes or 0 for no for the presence of each guideline.

Regarding individual guidelines, Figure 2 shows only 2 of 14 guidelines were adhered to by all 10 newspapers for both deaths. Notably, 6 guidelines were followed by less than one-third of newspapers, and 2 were not adhered to by any newspaper.

## Discussion

None of the 10 newspapers reviewed met the adherence threshold for high fidelity, highlighting widespread opportunities for improvement. As hypothesized, reporting on Bourdain’s suicide was more guideline adherent than reporting on Spade’s death. This suggests newspaper reporting on Bourdain’s suicide may have been influenced by the extensive criticism of the reporting on Spade’s suicide. Although most newspapers adhered to less than 50% of individual guidelines, our finding that 6 of 14 guidelines were followed rarely or never is concerning.

This study has limitations, including a focus on print rather than online newspaper articles and the selection of newspapers from large markets only. Also, we were unable to test for differences in adherence between national and regional newspapers.

In April 2014, Canadian journalists, the Mental Health Commission, and broadcasting corporations collaborated to establish reporting guidelines for suicide and create checklists for journalists to promote adherence.^6^ This resulted in increased adherence in the reporting on Robin Williams’s suicide in August 2014 (ie, 85% of articles had ≥70% adherence; 55% of articles had ≥80% adherence).^6^ A collaboration between US media staff, governmental agencies, and other stakeholders when updating and disseminating the recommended reporting guidelines for suicide may increase adherence, which in turn might reduce preventable harm.
